# Mapping Echocardiographic Practice in Emilia-Romagna: A Regional Healthcare Census

**DOI:** 10.3390/jcm15103719

**Published:** 2026-05-12

**Authors:** Andrea Barbieri, Francesca Mantovani, Francesca Bursi, Mattia Malaguti, Federico Fortuni, Luca Moderato, Ylenia Bartolacelli, Simone Binno, Alessandro Malagoli, Rita Pavasini, Chiara Pedone, Sergio Suma, Angelo Squeri, Alessandra Albini, Anna Chiara Vermi, Giovanna Di Giannuario, Marianna Laurito, Mauro Li Calzi, Alessandro Navazio, Antonella Moreo, Giovanni Di Salvo, Scipione Carerj

**Affiliations:** 1 Cardiology Division, Department of Biomedical, Metabolic and Neural Sciences, University of Modena and Reggio Emilia, Policlinico di Modena, 41125 Modena, Italy; olmoberg@libero.it; 2 Cardiology Unit, Azienda USL-IRCCS di Reggio Emilia, Viale Risorgimento 80, 42123 Reggio Emilia, Italy; marianna.laurito@ausl.re.it (M.L.); alessandro.navazio@ausl.re.it (A.N.); 3 Cardiology Unit, Health and Sciences Department, University of Milan, ASST Santi Paolo e Carlo, 20162 Milan, Italy; francescabursi@gmail.com; 4 U.O.C di Cardiologia, Ospedale Santa Maria Bianca, 41125 Mirandola, Italy; malagutimattia@gmail.com; 5 Cardiology and Cardiovascular Pathophysiology, S. Maria Della Misericordia Hospital, University of Perugia, 06121 Perugia, Italy; fortuni.ff9@gmail.com; 6 Cardiology Unit, ASST di Cremona, Ospedale Oglio-Po, 26041 Casalmaggiore, Italy; moderatoluca@gmail.com; 7 Department of Cardio-Thoracic and Vascular Medicine, Institute of Cardiology University of Bologna, Paediatric Cardiology and Adult Congenital Heart Disease Program, IRCCS Azienda Ospedaliero-Universitaria di Bologna, 40138 Bologna, Italy; ylenia.bartolacelli@gmail.com; 8 Unità di Ricerca Cardio-Renale ed Ipertensione, Università di Parma, 43126 Parma, Italy; s.binno@ausl.pc.it; 9 Division of Cardiology, Nephro Cardiovascular Department, Baggiovara Hospital, University of Modena and Reggio Emilia, 41125 Modena, Italy; ale.malagoli@gmail.com; 10 Cardiology Unit, Azienda Ospedaliero Universitaria di Ferrara, 44124 Ferrara, Italy; pvsrti@unife.it; 11 Cardiology Division, Maggiore Hospital, 40138 Bologna, Italy; chiara.pedone@ausl.bologna.it; 12 U.O. Cardiologia, Azienda Ospedaliera-Universitaria di Parma, 43126 Parma, Italy; sergiosuma.md@gmail.com; 13 Maria Cecilia Hospital, GVM Care & Research, 48033 Cotignola, Italy; 14 U.O Cardiologia, Ospedale Bufalini, 47521 Cesena, Italy; alessandra.albini@yahoo.it; 15 U.O. Cardiologia e UTIC, Ospedale Guglielmo da Saliceto, 29121 Piacenza, Italy; annachiara.vermi@hotmail.it; 16 U.O Cardiologia, Ospedale Infermi, 47923 Rimini, Italy; gdigiannuario@gmail.com; 17 U.O Cardiologia, Ospedale Santa Maria della Scaletta, Azienda USL di Imola, 40026 Imola, Italy; 18 Department of Cardiology, De Gasperis Cardio Center, ASST Grande Ospedale Metropolitano Niguarda, Piazza dell’Ospedale Maggiore 3, 20162 Milan, Italy; antonella.moreo@ospedaleniguarda.it; 19 Paediatric Cardiology Unit, Department of Child and Woman’s Health, University of Padua, 35128 Padua, Italy; giodisal@yahoo.it; 20 Cardiology Division, University Hospital Polyclinic G. Martino, University of Messina, 98125 Messina, Italy; scipione.carerj@unime.it

**Keywords:** census, echocardiography, echocardiographic activity, technology, echocardiographic laboratory, echocardiographic practice

## Abstract

**Highlights:**

**What are the main findings?**

- Echocardiography is a high-volume service, yet health-system-level data on laboratory organization and digital integration are often incomplete, limiting quality benchmarking and equity assessments.
- Voluntary surveys tend to overrepresent larger or academic centres and underrepresent smaller, private outpatient laboratories, biasing estimates of technology adoption and advanced-modality uptake.
- Core quality enablers (image archiving, structured reporting, EHR connectivity, adequate exam time, and advanced echocardiographic tools) are widely recommended, yet their real-world implementation across service networks remains unclear.

**What are the implications of the main findings?**

- An official-list-based regional census achieved high coverage (82%) among public and accredited private providers, offering a system-level view of echocardiography across a mixed public–private network.
- Only 31% of laboratories met at least 4 of 5 predefined structural–digital readiness domains (≥4/5), with major gaps in digital infrastructure (PACS, structured reporting, EHR/FSE integration) and uneven adoption of advanced imaging (strain/3D), revealing actionable quality deficits.
- In a highly developed healthcare context used as a “stress test” setting, teaching hospital status and a formally designated echocardiography lead independently predicted higher structural quality, indicating that leadership and digital infrastructure, rather than procedural volume, are the primary drivers of echocardiographic quality.

**Abstract:**

**Aims:** To assess echocardiographic practice within a regional healthcare system. The Emilia-Romagna region, a high-performing, digitally advanced context, was therefore used as a “stress test” setting in which observed heterogeneity is unlikely to be overestimated. **Methods:** A region-wide census of echocardiography laboratories collected data on governance, staffing, workflow, digital infrastructure, and imaging capabilities. A 5-item structural–digital readiness index (0–5) included: Picture Archiving and Communication System (PACS) archiving, structured reporting, Electronic Health Record (EHR) integration, availability of advanced echocardiographic tools, and an appointment slot for transthoracic echocardiography (TTE) of ≥20 min. High quality was defined as ≥4. Logistic regression identified independent predictors. **Results:** Of 148 centers, 122 (82%) responded, reporting 294,156 TTEs in 2023 (range < 500 to >15,000 per center); 46% were accredited private centers. Public institutions showed greater digital maturity than private centers (*p* < 0.001), with higher PACS availability and structured reporting. Overall, 86% reported ≥ 20 min per examination. Advanced modalities were unevenly distributed: left ventricular strain (50%), 3D imaging (33%), and stress echocardiography (42%). Workforce limitations were common, with 80% of centers lacking sonographers. A high structural–digital readiness index (score ≥ 4) was achieved by 38 laboratories (31%) and was associated with digital infrastructure and advanced imaging (*p* < 0.001). In multivariable analysis, university affiliation (OR 8.2–9.1) and a designated echocardiography lead (OR 4.1) independently predicted high quality, whereas procedural volume was not independently associated with quality. **Conclusions:** Marked variability in echocardiographic infrastructure and quality persists despite an advanced organizational and technological context. Leadership and digital infrastructure are the primary determinants of quality.

## 1. Introduction

Echocardiography remains one of the most widely used, cost-effective, and versatile imaging modalities in cardiovascular medicine [1]. Its applications range from initial diagnostic triage to procedural guidance and long-term patient monitoring [2]. Despite its widespread use, few health systems have systematically assessed the structure, distribution, and integration of echocardiographic services within regional or national care models. Mapping this variability is essential to ensure equitable access, uphold quality standards, and optimize resource allocation [3,4,5,6].

While previous surveys by the Italian Society of Echocardiography and Cardiovascular Imaging (SIECVI) [7,8,9,10,11,12] have provided valuable snapshots in Italy, these efforts have been limited by incomplete participation and underrepresentation of private centers. Real-world echocardiographic data are limited and primarily come from voluntary participation in international and national surveys, which may introduce several biases. To address this gap, we conducted a comprehensive census of echocardiographic laboratories in Emilia-Romagna (SIECVI-ERR), one of Italy’s most populous regions. Emilia-Romagna can be considered a high-performing [13] and digitally mature [14] healthcare system, and was therefore used as a ‘stress test setting’: the heterogeneity observed in this best-case scenario is likely to be at least as pronounced in less-resourced systems.

This study aimed to (1) characterize the heterogeneity of echocardiographic laboratory organization within a regional healthcare system, including variations in staffing, governance, and digital integration; (2) quantify the adoption and penetration of advanced echocardiographic modalities (e.g., three-dimensional [3D], strain, stress echo), highlighting disparities across settings; and (3) identify modifiable barriers to high-quality practice and areas for potential intervention, including time constraints, perceived appropriateness of referrals, and the use of sonographers.

## 2. Methods

### 2.1. Italian Healthcare System and Organization of Echocardiographic Services

Italy has a universal, publicly funded healthcare system, known as ‘Servizio Sanitario Nazionale’ (SSN), which provides diagnostic and therapeutic services through a mixed network of public and private providers. Private centers are authorized by regional health authorities to deliver services reimbursed by the National Health Service (NHS), while regional health trusts directly manage public hospitals. In Emilia-Romagna, both public and private facilities fall under regional planning and are responsible for providing standardized healthcare services. However, these centers may differ in staffing models, infrastructure, and digital integration. In this survey, private echocardiographic laboratories are defined as facilities formally recognized by the Regional Health System to perform reimbursed cardiovascular ultrasound exams [15].

This administrative recognition should not be confused with scientific or technical certification by professional societies.

### 2.2. Electronic Health Records—Fascicolo Sanitario Elettronico (FSE)—And FSE-Enabled Report Delivery

The Italian *Fascicolo Sanitario Elettronico* (FSE) is a citizen-centered EHR system that collects and makes available digital health documents generated across the healthcare system. It is implemented regionally and is supported by national interoperability. In this survey, “FSE-enabled report delivery” was defined as the laboratory’s capability to publish the final, validated report to the patient’s FSE via an electronic feed from the local reporting system to the regional FSE platform, in accordance with consent/opt-out settings. This workflow is relevant because diagnostic laboratories are major producers of clinical documentation, and FSE delivery facilitates continuity of care, and cross-setting information sharing while reducing fragmented or duplicate reporting [16].

### 2.3. The Role of SIECVI in Echocardiographic Standardization

This survey has received endorsement from the Italian Society of Echocardiography and Cardiovascular Imaging (SIECVI), the national reference for training, certification, and quality promotion in cardiovascular ultrasound. SIECVI offers voluntary accreditation programs aligned with international standards, including those of the European Association of Cardiovascular Imaging (EACVI), to promote structured training, organizational quality, and diagnostic excellence. These certifications, although not mandatory for regional reimbursement, serve as a recognized benchmark for echocardiographic quality.

### 2.4. Study Design and Data Collection

Between January and December 2024, the regional delegate (FM) coordinated provincial delegates to identify and contact all echocardiography service providers in the Emilia-Romagna regional healthcare system (*n* = 148) (Supplementary Materials S1), including 90 private and 58 public facilities, using the official list of public and private centers provided by the Regional Health Authority. Centers were contacted by email and phone to explain the research purpose and provide a link to the survey. Periodic reminders were sent to maximize response rates and data completeness. The survey was available from September 2024 to February 2025.

All centers were asked to complete a structured electronic questionnaire. The questionnaire included 75 items covering key domains of echocardiographic practice, including facility characteristics, staffing and infrastructure, digital systems, organization and access to care, activity volume and case mix, availability of advanced and quantitative imaging techniques, software resources, perceived examination time, scientific engagement and training needs, and the presence of a formally designated echocardiography lead (Supplementary Materials S2). A ‘designated echocardiography lead’ was defined as a formally identified physician responsible for echocardiography activities in the laboratory; the questionnaire did not further distinguish among managerial, clinical, or educational functions.

Data were collected electronically using a standardized questionnaire completed by a cardiologist at each regional echocardiography laboratory.

We constructed a 5-item structural–digital readiness index (0–5) to summarize the co-occurrence of widely recommended quality-enabling features. Each component was weighted equally by design, as the score was intended as a pragmatic descriptive framework rather than a validated performance or prognostic index. Each item represents a distinct, non-hierarchical domain of echocardiographic quality recommended by international societies [17], and no external evidence currently supports differential weighting among these domains. The included items were: (1) availability of a Picture Archiving and Communication System (PACS) or an equivalent system for systematic archiving and retrieval of echocardiographic images; (2) structured digital reporting; (3) FSE-enabled report delivery; (4) an appointment slot for TTE of at least 20 min; and (5) availability of advanced echocardiographic analyses (strain and/or 3D imaging).

Each criterion was scored as 1 if present and 0 if absent. The total score ranged from 0 to 5. A cut-off score of ≥4 was set a priori to identify laboratories that met most of the predefined structural–digital readiness domains (Table 1).

### 2.5. Statistical Analyses

Continuous and ordinal variables were summarized using medians (interquartile ranges, IQR) and compared using the Mann–Whitney U test; categorical variables were compared using the χ^2^ test. For descriptive purposes, the number of sonographers was analyzed both as a binary variable (presence vs. absence) and, among centers with sonographers, as a continuous count. A two-sided *p*-value < 0.05 was considered statistically significant.

Procedural volume was analyzed as an organizational characteristic reflecting service activity, rather than as a surrogate measure of clinical quality.

To identify external organizational factors associated with high structural quality, we fitted a multivariable logistic regression model with the binary structural–digital readiness index (≥4 vs. <4) as the dependent variable. Sensitivity analyses used alternative definitions of structural–digital readiness, including a stringent definition (score = 5) and a more permissive definition (score ≥ 3). To avoid conceptual overlap with the index, only contextual and governance variables not included in the index were entered as covariates: public versus private accredited center, university teaching status (main campus or branch vs. non-teaching hospital), presence of a formally designated echocardiography lead, and membership in at least one scientific society. Odds ratios (OR), 95% confidence intervals (CI), and *p*-values were reported; model fit and multicollinearity were assessed, and all variance inflation factors were <3. Procedural volume correlated strongly with teaching hospital status and was therefore excluded from the adjusted model to prevent collinearity. Its association with the structural–digital readiness index is presented only in univariate analyses.

Statistical analyses were performed using JMP^®^ Pro 18.0.2 (SAS Institute Inc., Cary, NC, USA). A two-sided *p*-value < 0.05 was considered statistically significant.

## 3. Results

### 3.1. Center Characteristics and Participation

**Figure 1:**
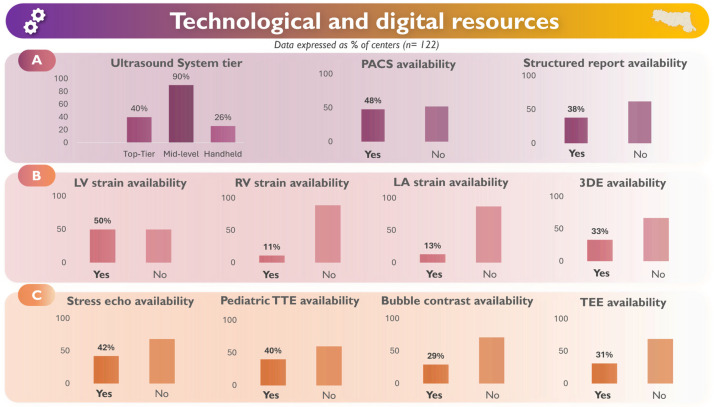
Technological and digital resources of echocardiography laboratories in Emilia-Romagna. Panel **A**: ultrasound systems and digital infrastructure. Panel **B**: advanced imaging tools. Panel **C**: advanced procedures. Data are presented as percentages of centers.

All activity data were based on local self-reports. Of 148 identified centers, 122 (82%) returned complete datasets. Fifty-six (46%) were private institutions (accredited private centers). A comprehensive graphical summary of the main findings is shown in Figure 1.

### 3.2. Procedural Volume and Examination Types

In 2023, participating centers reported a cumulative self-reported volume of 294,156 TTEs. In total, 79% of centers reported predominantly outpatient activity (≥50% of examinations booked through the regional healthcare system), whereas 21% offered mainly inpatient or emergency echocardiographic services. Annual echocardiographic study volumes ranged from <500 to >15,000 per center.

**Figure 2:**
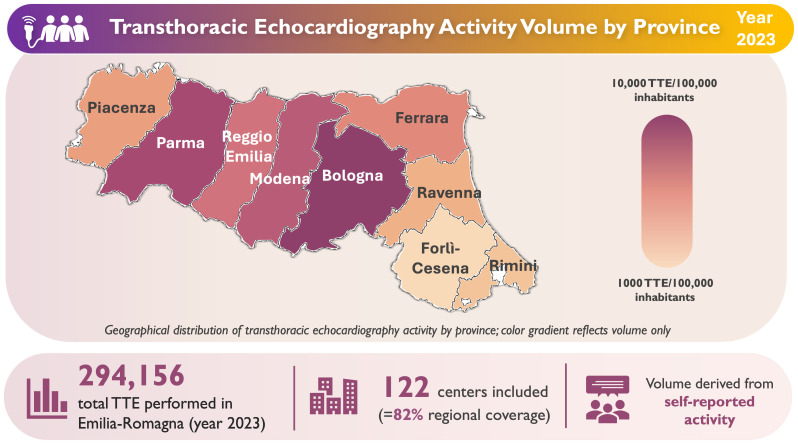
Transthoracic echocardiography (TTE) activity by province in Emilia-Romagna (2023). Annual TTE volume per 100,000 inhabitants, estimated from self-reported monthly activity (×12) and standardized to provincial population data (ISTAT 2023).

The number of TTEs performed per 100,000 inhabitants varied significantly across provinces, highlighting substantial territorial disparities in the regional provision of echocardiographic services (Figure 2). Centers were stratified by annual echocardiographic volume into three activity groups: low (<2000 studies/year), medium (2000–4000 studies/year), and high (>4000 studies/year). The threshold of 4000 examinations per year was selected a priori based on EACVI accreditation standards and national SIECVI survey data, reflecting the expected workload of a fully operational echocardiographic laboratory.

Thirty-seven out of 122 centers (31%) reported dedicated TEE activity (52% in public hospitals and 7% in private centers; *p* < 0.0001); three centers did not respond.

Pediatric echocardiography was performed at 48 centers (40%; 1 non-responder), totaling 8316 examinations annually. Among centers performing pediatric echocardiography, 79% did not provide services for all pediatric age groups, including neonatal patients.

### 3.3. Technological Resources

Technological resources varied widely across centers. Mid-level ultrasound systems were nearly universal (90%), whereas only 40% operated at least one top-tier platform and 26% used handheld devices. PACS availability was limited (48%), particularly in private institutions (33% vs. 59% in public hospitals). Structured digital reporting was implemented in 38% of laboratories. Among the remaining 62% without structured reporting, 31% used free-text digital reports, and 31% relied on other non-structured formats (e.g., non-templated digital reports or non-digital reporting).

Overall, 67% of centers reported FSE-enabled electronic health record (EHR) integration, enabling the direct delivery of validated echocardiographic reports into the regional EHR.

Advanced post-processing capabilities showed similarly uneven diffusion. Strain analysis was available for the left ventricle in 50% of centers, but for the right ventricle (11%) and the left atrium (13%), it was rarely available, and right atrial strain was virtually unused [18].

Three-dimensional TTE was implemented in one-third of centers. Among laboratories equipped with at least one high-end platform, 85% lacked at least one core advanced analysis package, and only 15% had the full suite.

Stress echocardiography was available in 42% of laboratories, though procedural activity remained modest across modalities, with typical monthly volumes of 1–5 examinations. Pediatric echocardiography was offered in 40% of centers, with low-to-moderate monthly volumes (median 20), indicating institutional diversification rather than a defining structural characteristic.

Bubble-contrast studies were performed at 31 centers, with a median of 2 [2–5] examinations per month. Additional specialized procedures showed low activity: transcranial Doppler at 13 centers (2 [1–7] studies per month) and TEE at 37 centers (16 [8–32] per month).

### 3.4. Human Resources and Organization

The median number of cardiologists per laboratory was 4 (IQR 2–6), slightly higher in public hospitals than in accredited private centers. A formal leadership structure was inconsistently present: 26.5% of centers had a designated director, and 52% had a formal lead. Most echocardiographers (59%) spent most of their working time in the laboratory, although only 10% had a workload predominantly dedicated to echocardiography (>75%).

TTE activity was generally higher in public centers (172 [IQR 62–394] vs. 120 [60–190] studies per month). However, the operator-to-exam ratio was similar across settings (≈50 examinations per operator per month), though it was more variable in public institutions.

Dedicated sonographers were uncommon: 80% of centers had none, 12% had one, and only a small minority had two or more. Their distribution did not differ significantly between public and private laboratories. Sonographers were more common in high-volume centers (29% vs. 13%). Among centers employing at least one sonographer, telecardiology via digital archiving systems was reported in approximately half of the cases.

### 3.5. Appropriateness and Workflow

**Figure 3:**
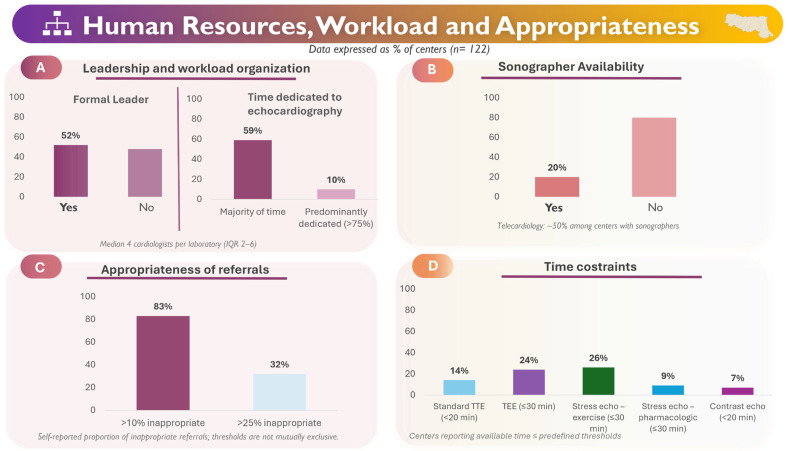
Human resources, workload, and appropriateness in echocardiography laboratories. Panel **A**: leadership and time allocation. Panel **B**: sonographer availability and telecardiology. Panel **C**: perceived referral inappropriateness. Panel **D**: time constraints across modalities. Data are expressed as percentages of centers.

An estimated 83% of centers reported the perception that more than 10% of their echocardiographic referrals were clinically inappropriate, and 32% reported that more than 1 in 4 referrals were considered clinically inappropriate (Figure 3). Although the overall prevalence did not differ significantly between public and private institutions, the distribution of responses revealed distinct patterns: moderate levels of inappropriateness (around 25%) were more frequently reported at public centers, whereas private facilities more commonly reported rates of 50% or higher. Time constraints were also widely reported.

For standard transthoracic echocardiography (TTE), 14% of centers reported examination times < 20 min, while 20 min was the most commonly reported duration, resulting in 86% of centers allocating ≥ 20 min overall. No relevant differences were observed between public and private centers. For contrast-enhanced TTE, examination times were generally longer than for standard TTE, with 93% of centers allocating at least 20 min.

Given differences in procedural complexity, time allocation was assessed using modality-specific thresholds. Among centers performing transesophageal echocardiography (TEE), 24% reported examination times of ≤30 min. Similar patterns were observed for stress echocardiography: 26% of centers performing exercise stress echocardiography and 9% performing pharmacological stress echocardiography reported examination times of ≤30 min.

Given the limited number of private centers performing TEE, stress, or contrast echocardiography, comparative analyses between public and private institutions were restricted to standard TTE.

### 3.6. Accreditation, Education, and Scientific Engagement

Only 16 centers (11%) were SIECVI- or EACVI-accredited, and 31% hosted residents or structured educational programs. The most frequently requested initiatives were workshops on advanced modalities, such as speckle-tracking and 3D echocardiography, as well as interactive case-based sessions. Scientific participation was equally limited: only a minority of respondents reported membership in national or international cardiovascular imaging societies, suggesting limited access to continuing professional development and best-practice updates. This pattern points to a gap in training and scientific integration, particularly in smaller or peripheral laboratories.

### 3.7. Profiles of Echocardiography Laboratories According to Structural–Digital Readiness Index

**Figure 4:**
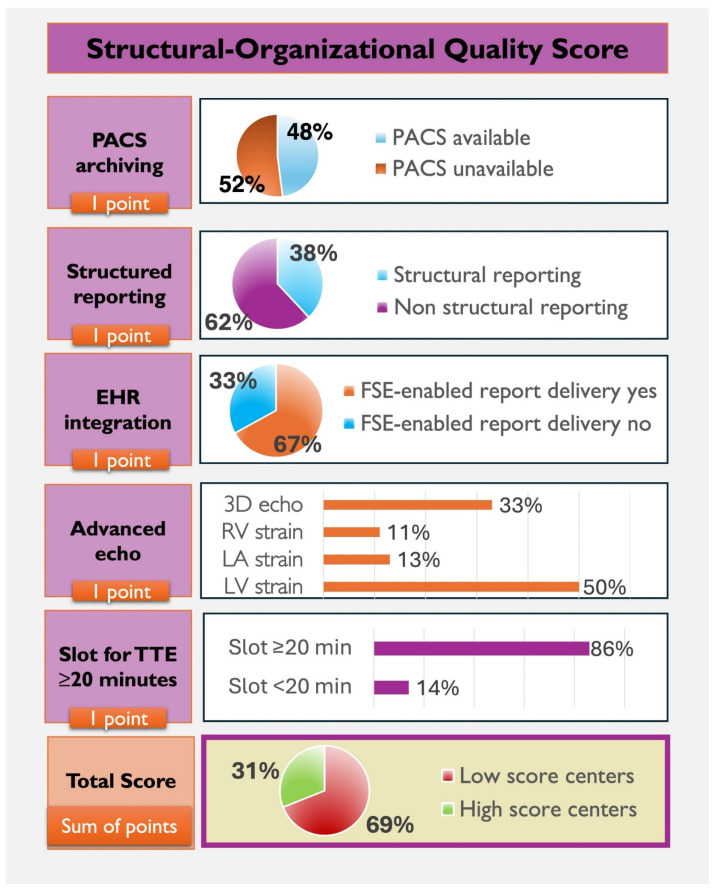
Structural–digital readiness index across echocardiography laboratories. Structural–digital readiness index (*n* = 122). The 5-item score (0–5) includes PACS, structured reporting, EHR integration, advanced imaging (3D/strain), and TTE time ≥ 20 min. Charts show the proportion meeting each criterion and the overall score distribution; ≥4 indicates fulfillment of most domains.

Score distributions ranged from 0 to 5, with a gradual spread across categories, supporting the index’s use as a descriptive, non-dichotomous measure. In total, 31% of centers achieved a score ≥ 4, and only 13% fulfilled all five domains (Figure 4). Sensitivity analyses using alternative thresholds (score = 5 and score ≥ 3) yielded directionally consistent results, with academic affiliation and designated echocardiography leadership remaining the strongest correlates of higher structural–digital readiness (Supplementary Materials S2, Table). Major gaps were observed in PACS availability (48%) and structured reporting (38%), whereas 86% of laboratories reported scheduled TTE slots ≥ 20 min. As summarized in Table 2, high-scoring laboratories differed consistently from low-scoring centers across governance, digital infrastructure, activity profile, and workforce. High-quality centers were more often public institutions (76% vs. 44%, *p* = 0.0013; OR 3.9) and teaching hospitals (26% vs. 3%, *p* < 0.0001; OR 18.0), with higher accreditation rates (29% vs. 7%, *p* = 0.001) and a designated echocardiography lead more often (50% vs. 14%, *p* < 0.0001; OR 6.0).

Clinical activity also differed substantially. High-score centers were more likely to exceed 4000 TTE/year (41% vs. 11%, *p* = 0.0002; OR 5.7) and to perform advanced procedures, including contrast (53% vs. 7%, *p* < 0.0001; OR 14), pediatric echocardiography (58% vs. 31%, *p* = 0.006; OR 3.0), TEE (74% vs. 11%, *p* < 0.0001; OR 22), stress echocardiography (46% vs. 7%, *p* = 0.0003; OR 10.6), and structural heart imaging (45% vs. 2%, *p* < 0.0001). Workforce differences mirrored these patterns, with more cardiologists dedicated to echocardiography (4.7 ± 2.7 vs. 2.8 ± 2.1, *p* = 0.0004; OR 1.4 per unit increase) and a higher rate of sonographer employment (32% vs. 14%, *p* = 0.026). By contrast, perceived critical issues (technological inadequacy, inappropriate referrals, shortage of personnel, insufficient time per examination, and limited access to training) did not differ significantly between high- and low-quality laboratories (all *p* > 0.27).

In multivariable analysis (Table 3), both primary and branch university teaching hospitals (ORs 8.2 and 9.1, respectively) and the presence of a designated echocardiography lead (OR 4.1) independently predicted high structural quality. Public governance showed a non-significant positive trend (OR 2.6, *p* ≈ 0.07), whereas membership in scientific societies was not associated with higher scores.

## 4. Discussion

Emilia-Romagna is consistently ranked among the highest-performing healthcare systems in Europe, placing first in Italy and among the top regions for healthcare quality and efficiency [13]. In this context, it can be viewed as a best-case scenario within a publicly funded European healthcare model. However, this first regional census of echocardiographic practice in a mixed public–private system reveals marked heterogeneity in organization and delivery across centers, including staffing, leadership, workflow, and the availability of advanced modalities such as strain, three-dimensional, and stress echocardiography. The persistence of substantial variability in digital integration, reporting standards, and access to advanced imaging, even in such a setting, underscores the broader relevance of our findings. Although these findings should be interpreted in light of the self-reported nature of several organizational, technological, and workflow-related variables, they suggest that similar disparities are likely to be amplified in less-resourced or less digitally integrated regions.

### 4.1. Pediatric Echocardiography: A Niche Service in Non-Pediatric Cardiology Laboratories

Pediatric echocardiography contributed only marginally to the regional network’s overall organizational profile. Although available in 40% of centers, activity volumes were modest, and their distribution largely reflected pre-existing institutional characteristics rather than serving as a defining structural feature. As such, pediatric imaging should be viewed as one of several ancillary modalities whose presence signals broader service diversification, but it does not substantially influence the overall assessment of infrastructural maturity or digital readiness across laboratories.

### 4.2. Technological and Digital Gaps

Digital and technological resources varied widely. While mid-level ultrasound systems were nearly universal, high-end platforms and advanced post-processing tools were concentrated in a minority of structurally mature centers. Digital infrastructure remained a significant limitation, with approximately two-thirds of laboratories lacking PACS, structured reporting, or integrated EHR connectivity. These deficiencies constrain workflow quality and limit the deployment of advanced imaging or AI-enabled tools [17].

### 4.3. System-Wide Heterogeneity in Echocardiographic Structure

**Figure 5:**
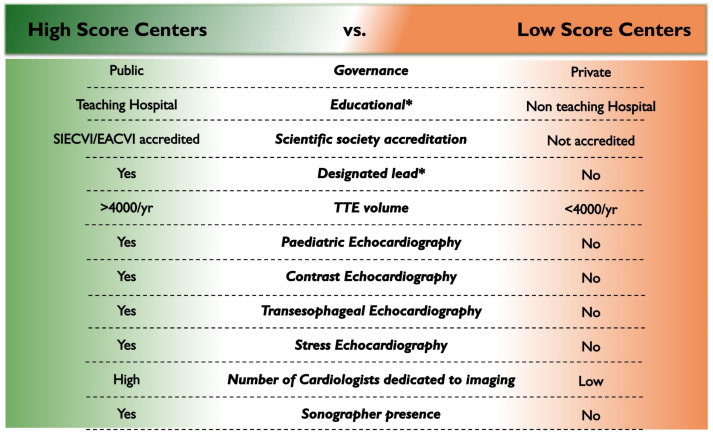
Organizational and operational characteristics of high- vs. low-readiness laboratories. Comparison of laboratories with high (≥4) versus low (<4) structural–digital readiness index. Summary of organizational, academic, and operational characteristics. Asterisks indicate variables independently associated with higher readiness.

The structural–digital readiness index we proposed should be viewed as a pragmatic descriptor, based on an a priori, partially arbitrary combination of domains that reflect quality-enabling organizational and infrastructural features, rather than as a direct measure of clinical quality. While the index captures conditions that may support high-quality practice, it does not directly assess diagnostic accuracy, referral appropriateness, or patient outcomes. The ≥4 threshold should not be interpreted as a validated definition of quality, but rather as a pragmatic criterion for identifying laboratories that meet most of the predefined readiness domains. Despite these limitations, the index provided a structured representation of system-level heterogeneity, revealing marked variability across the regional network (Figure 5). Only one-third of laboratories met the ≥4 threshold, reflecting fulfillment of most predefined domains without implying equivalence across different item combinations, particularly with respect to digital infrastructure, workflow organization, and access to advanced imaging. Public and teaching hospitals operated with more mature systems and clearer organizational models than accredited private centers. Although the availability of high-end ultrasound platforms contributed to this gradient, the key differentiators were the presence of a formal echocardiography lead and overall institutional maturity, identified as the only independent predictors of high structural quality. Because the survey did not distinguish the specific operational functions of this role, the observed association should be interpreted as reflecting formal organizational designation rather than a single standardized leadership model. Notwithstanding these findings, the landscape depicts one in which echocardiographic quality depends strongly on institutional readiness rather than on equipment availability alone.

### 4.4. Current Integration of Sonographers in the Regional Echocardiographic Network

Our survey indicates that the integration of sonographers into echocardiographic practice in Emilia-Romagna remains limited, with only modestly higher adoption in public institutions. Overall, 21% of centers reported employing at least one sonographer, with a mean of 0.4 per center. Among laboratories with sonographers, the median was 1 (IQR 1–2). This pattern reflects a predominantly physician-driven model of echocardiography, in contrast to many international settings where sonographers play a central role in image acquisition and preliminary interpretation [18]. The limited availability of sonographers highlights a key workforce constraint, with implications for workflow sustainability and physician workload. Alternative staffing models, including greater integration of trained sonographers and task redistribution, could support service delivery while remaining aligned with European training and accreditation frameworks.

### 4.5. Echocardiographic Support for Structural Heart Interventions: Current Regional Availability

The expansion of percutaneous structural interventions has reinforced the role of echocardiographic guidance. In our survey, only a minority of centers (≈15–20%) provided structured support for interventional procedures, and this support was largely concentrated in high-volume public hospitals. Most accredited private centers were not involved and lacked dedicated personnel or intra-procedural TEE capability. This pattern likely reflects differences in volume, resources, and integration with interventional cardiology teams. Procedural echocardiography requires advanced training, high operator expertise, and immediate availability [18,19], conditions difficult to ensure in non-tertiary or outpatient-based settings. Given the projected expansion of structural interventions, regional healthcare planning should aim to identify referral centers with certified echocardiographic support, integrated referral pathways, and dedicated training and resources.

### 4.6. Time for Echocardiographic Examinations: Implications for Quality

Reported examination times highlight key constraints in echocardiographic practice. Most centers allocated ≥ 20 min for standard TTE, though a minority reported shorter slots. For more complex modalities, about one quarter of centers reported TEE durations of 30 min, with similar patterns for stress echocardiography. These estimates reflect organizational factors, including workflow, staffing, and throughput requirements, that shape examination scheduling. Such constraints contrast with international accreditation standards [20], including EACVI recommendations, which emphasize adequate time to ensure diagnostic accuracy and complete documentation [17,21,22,23]. In comparison, a recent British survey reported an average of 40 min for standard TTE [3]. Evidence links time pressure to suboptimal image acquisition, reduced interpretive accuracy, and incomplete adherence to advanced protocols [3,24,25]. Perceived time inadequacy should therefore be treated as a potential quality alert, prompting a review of organizational models, reimbursement structures, and staffing ratios to align examination time with the complexity of contemporary echocardiographic practice.

### 4.7. Referral Inappropriateness: A Persistent and Underestimated Issue

Self-reported perceptions of referral inappropriateness emerged as a recurring concern. Although the survey was not designed to measure appropriateness objectively, these perceptions reflect a frequent mismatch between clinical questions and TTE’s diagnostic value. Similar concerns have been highlighted in previous national and international audits [26,27,28,29], in which inappropriate or low-yield requests accounted for a substantial proportion of TTE examinations and were associated with workflow inefficiencies and reduced diagnostic impact [30]. The variability reported across centers likely reflects local referral practices, limited triage mechanisms, and the absence of structured pathways for common clinical scenarios [5]. In systems without dedicated echocardiography leadership or formalized booking criteria, clinicians may often request TTE as a default diagnostic test, even when alternative tests or clinical follow-up may be more appropriate [31]. Although inappropriate testing was not objectively assessed, its consistent perception across centers suggests a system-level issue. Structured referral criteria, improved communication, and decision-support tools may help reduce low-value testing and optimize resource use [32].

### 4.8. Comparison with Previous National and International Echocardiography Data

Unlike previous SIECVI national surveys [7,8,9,10,11,12], which relied on voluntary participation from known or affiliated laboratories, our census used an exhaustive list of all public and private accredited centers provided by the Regional Health Agency, yielding a nearly 80% response rate. This systematic approach captured smaller, community-based, and lower-volume private institutions. These facilities are often underrepresented in national registries, providing a more realistic picture of infrastructure and access to advanced technology.

Previous national surveys provided valuable insights but lacked denominator data on population coverage. In our dataset, 42% of laboratories performed stress echocardiography, compared with 79% in the latest SIECVI report [9], likely reflecting differences in case mix, available resources, and referral networks. Similarly, the 2022 SIECVI survey reported that 3D and speckle-tracking imaging were available in 75% and 80% of centers, respectively [8], indicating lower adoption rates and greater public–private heterogeneity. Image-archiving deficiencies, particularly in private centers, emerged as a significant, previously underreported gap.

Our results complement those from the EACVI [33,34] and British Society of Echocardiography surveys [35], which primarily included tertiary or accredited centers with greater resource availability, specialized staff, and stronger adherence to guideline-based protocols. This selection bias likely explains the higher reported use of advanced echocardiography in those studies compared with our broader regional snapshot. These comparisons should be interpreted with caution, because differences in sampling frames, response behavior, accreditation status of participating centers, and variable definitions may substantially affect comparability.

### 4.9. Perceived Criticalities, Training Needs, and System-Level Expectations

The survey identified key barriers to effective echocardiographic practice, including limited examination time, suboptimal structured reporting, insufficient training, and restricted access to advanced imaging. These issues were consistently reported across centers, supporting a system-level origin. Training needs were greatest for advanced modalities and for standardizing measurements. Respondents emphasized SIECVI’s role in promoting networking, certification, and structured training, and suggested defining minimal technical standards, developing shared digital platforms, and implementing regional referral pathways.

### 4.10. A Paradox of Innovation and Inertia

Despite growing enthusiasm for artificial intelligence in cardiovascular imaging [36,37], our survey highlights persistent foundational gaps. Many centers still lack essential digital infrastructure, adequate reporting systems, and sufficient time to support high-quality examinations. Our findings reinforce the idea that digital innovation and AI-based tools cannot replace the organizational foundations of echocardiographic services, including adequate workflow design, professional expertise, training, and institutional governance. This creates a paradox in which innovation risks benefiting only a minority of structurally mature institutions, potentially widening disparities rather than reducing them [38]. Addressing these limitations requires coordinated planning, governance, and investment in human resources, not only in technological upgrades.

## 5. Limitations

Several limitations should be considered when interpreting the findings, which reflect organizational and structural patterns rather than validated measures of clinical quality.

This survey relied on self-reported data from participating laboratories, which may introduce reporting bias, particularly for subjective measures such as perceived examination appropriateness and reported time per procedure. Although not objectively quantified, these measures capture critical aspects of workflow and quality that are otherwise difficult to assess using administrative data [39,40]. Furthermore, although unlikely to substantially alter the conclusions, a small fraction of TTE may have occurred outside the regional health system and thus may not have been captured in the dataset.

Our analysis is based on a single regional healthcare system, which may limit direct generalizability. However, it can be viewed as a ‘stress test’ of an advanced setting, in which the observed heterogeneity is unlikely to be overestimated and may underestimate gaps in less mature systems.

Our census reflects laboratory organization and practices in calendar year 2023; given the rapid evolution of Italy’s digital health landscape, the current situation may differ. Over the past two years, implementation has accelerated due to investments from the National Recovery and Resilience Plan (PNRR; NextGenerationEU-funded), which supports the expansion and standardization of the Fascicolo Sanitario Elettronico (FSE) and broader healthcare digital transformation, including technology renewal in hospitals [41]. Therefore, our results should be interpreted as a baseline snapshot. Notably, the 2023 census provides a framework for periodic reassessment, enabling us to quantify progress and evaluate the real-world impact of PNRR-supported measures on echocardiography laboratories.

Finally, although the response rate was high, non-response bias cannot be ruled out. Non-responding centers were predominantly small, non-academic laboratories, with a higher proportion of accredited private outpatient facilities and no clear geographic clustering. However, detailed information on examination volume, workflow, and digital infrastructure was unavailable for these centers.

## 6. Conclusions

This study is the first region-wide census to comprehensively characterize echocardiographic practice in a mixed public–private healthcare system. The findings reveal marked structural and digital heterogeneity across laboratories, with substantial variability in workflow organization, technological maturity, and access to advanced imaging. Importantly, structural quality was determined primarily by institutional maturity, particularly teaching hospital status, and by the presence of formal echocardiography leadership. In this regional observational setting, institutional leadership and digital infrastructure were key correlates of higher structural–digital readiness, with implications for imaging governance, accreditation strategies, and health-system planning across diverse healthcare settings.

## Figures and Tables

**Table 1:** Definition of the structural-organizational structural–digital readiness index.

Item	Definition/Criteria for Scoring = 1	Rationale/Reference to EACVI Standards
PACS	Availability of a PACS.	Ensures traceability, reproducibility, and quality control of echocardiographic data.
Appointment slot for TTE ≥ 20 min	Median examination time per transthoracic echocardiogram ≥ 20 min.	Reflects adequate time for comprehensive assessment and reporting.
Structured digital reporting	Availability of standardized, template-based digital echocardiographic reports (structured reporting).	Promotes standardization, completeness, and readability of echocardiographic documentation.
Access to Electronic Health Records (EHR) (FSE-enabled report delivery)	Direct access to patients’ electronic health records allows integration of echocardiographic findings with clinical data.	Facilitates multidisciplinary care, data continuity, and adherence to digital health standards.
Availability of advanced echocardiographic analyses (strain and/or 3D imaging)	Presence of strain analysis and/or three-dimensional echocardiographic capabilities, either on-cart or workstation-based.	Indicates technological readiness for advanced quantitative imaging as per the EACVI advanced standard.

Scoring and Interpretation: Each criterion is scored as 1 if present and 0 if absent. The total index ranges from 0 to 5. A composite score ≥ 4/5 indicates that laboratories meet the threshold for adherence to good echocardiographic practice standards. Abbreviations. TTE: Transthoracic Echocardiography; PACS: Picture Archiving and Communication System; EHR: Electronic Health Record; EACVI: European Association of Cardiovascular Imaging.

**Table 2:** Characteristics of centers among total study participants, stratified by the structural–digital readiness index; univariate logistic regression analysis showing the Odds ratio (95% CI) for a high (≥4/5) score.

	Total N = 122	Low Score N = 84	High Score N = 38	*p*	OR (95% CI) for High Score (≥4/5)	*p*
Public Center	69%	44%	76%	0.0013	3.9 (1.7–9.8)	0.001
University training center: - Main - Branch - No	10% 17% 72%	3% 8% 88%	26% 37% 37%	<0.0001	18 (5–86) 10 (4–33) 1	<0.0001 <0.0001
Accreditation to SIECVI or EACVI	14%	7%	29%	0.0013	5.3 (1.8–16.7)	0.002
Presence of a designated responsible	25%	14%	50%	<0.0001	6.0 (2.5–14.9)	<0.0001
Number of echo boxes, median (Q1–Q3)	2 (1–2)	1 (1–2)	2 (2–3)	<0.0001	1.30 (1.03–1.78)	0.0028
Number of cardiologists on the team, mean ± SD	3.5 ± 2.5	2.8 ± 2.1	4.7 ± 2.7	0.0004	1.4 (1.2–1.6)	0.0003
Presence of sonographers	20%	14%	32%	0.026	2.8 (1.1–7.0)	0.030
Number of top-level ultrasound machines (with 3D probes and advanced software): - None (reference) - One - Two - Three or more	60% 21% 11% 8%	81% 14% 4% 2%	18% 37% 26% 18%	<0.0001	1 10.8 (3.7–34.3) 30.9 (7.6–165.9) 32 (6.4–250)	<0.0001 <0.0001 <0.0001
Number of mid-level ultrasound machines, median (Q1–Q3)	1 (1–2)	1 (1–2)	2 (1–3)	0.0003	2.0 (1.4–3.0)	0.0002
Number of portable ultrasound machines, median (Q1–Q3)	0 (0–1)	0 (0–0)	0 (0–1)	0.0002	2.4 (1.6–4.0)	<0.0001
How much time doesokok the team dedicate to imaging activities? - Predominantly (>75%); - Majority (50–75%); - Minor (25–50%); - Occasionally (<25%) (reference)	10% 47% 39% 3%	9% 43% 44% 4%	10% 58% 29% 3%	0.414	0.1 (0.1–35.9) 1.8 (0.2–38.3) 0.9 (0.1–19.0) 1	0.752 0.595 0.925
Volume of transthoracic echocardiograms greater than 4000 per year	20.5%	10.7%	40.5%	0.0002	5.7 (2.2–15.9)	0.0003
Supra-aortic trunk Doppler ultrasounds performed	41%	43%	37%	0.459	1.3 (0.6–2.9)	0.497
Bubble-contrast echocardiograms performed	29%	13%	63%	<0.0001	11 (5–29)	<0.0001
Contrast-enhanced echocardiograms performed	22%	7%	53%	<0.0001	14 (5–43)	<0.0001
Exercise stress echocardiography performed	19%	7%	46%	0.0003	10.6 (3.9–32.8)	<0.0001
Dipyridamole stress echocardiography performed	24%	8%	58%	<0.0001	17.0 (6.2–52.6)	<0.0001
Coronary flow reserve assessment performed	9%	5%	18%	0.017	4.4 (1.2–17.8)	0.022
Dobutamine stress echocardiography performed	27%	9%	66%	<0.0001	20.2 (7.7–60.6)	<0.0001
Transcranial Doppler ultrasound performed	12%	9%	18%	0.123	2.4 (0.7–7.5)	0.134
Pediatric TTE performed	40%	31%	58%	0.006	3.0 (1.4–6.8)	0.006
TEE performed	31%	11%	74%	<0.0001	22 (8–64)	<0.0001

Abbreviations: TTE: Transthoracic Echocardiography; OR: Odds Ratio; CI: Confidence Interval.

**Table 3:** Independent predictors of a high structural–digital readiness index score.

Variable	Adjusted OR	95% CI	*p*-Value
Public vs. private center	1.7	0.5–5.2	0.3150
Teaching status			
University main vs. no	8.2	1.8–45.7	0.0054
University branch vs. no	9.1	2.8–33.6	0.0002
Designated echocardiography lead	4.1	1.4–12.0	0.0081
Member of a scientific society	2.1	0.7–7.0	0.2100

Abbreviations: OR: Odds Ratio; CI: Confidence Interval.

## Data Availability

Data are property of SIECVI and are not available.

## Supplementary Materials

The following supporting information can be downloaded at: https://www.mdpi.com/article/10.3390/jcm15103719/s1.

## References

[1] Kisslo J. (2024). Echocardiography, the AHA, and 100 Years. Circulation.

[2] Cheitlin M.D., Armstrong W.F., Aurigemma G.P., Beller G.A., Bierman F.Z., Davis J.L., Douglas P.S., Faxon D.P., Gillam L.D., Kimball T.R. (2003). ACC/AHA/ASE 2003 Guideline Update for the Clinical Application of Echocardiography: Summary article. A report of the American College of Cardiology/American Heart Association Task Force on Practice Guidelines (ACC/AHA/ASE Committee to Update the 1997 Guidelines for the Clinical Application of Echocardiography). J. Am. Soc. Echocardiogr..

[3] Galderisi M., Cosyns B., Edvardsen T., Cardim N., Delgado V., Di Salvo G., Donal E., Sade L.E., Ernande L., Garbi M. (2017). Standardization of adult transthoracic echocardiography reporting in agreement with recent chamber quantification, diastolic function, and heart valve disease recommendations: An expert consensus document of the European Association of Cardiovascular Imaging. Eur. Heart J. Cardiovasc. Imaging.

[4] Lopez L., Saurers D.L., Barker P.C., Cohen M.S., Colan S.D., Dwyer J., Forsha D., Friedberg M.K., Lai W.W., Printz B.F. (2024). Guidelines for Performing a Comprehensive Pediatric Transthoracic Echocardiogram: Recommendations From the American Society of Echocardiography. J. Am. Soc. Echocardiogr..

[5] American College of Cardiology Foundation Appropriate Use Criteria Task Force, American Society of Echocardiography, American Heart Association, American Society of Nuclear Cardiology, Heart Failure Society of America, Heart Rhythm Society, Society for Cardiovascular Angiography and Interventions, Society of Critical Care Medicine, Society of Cardiovascular Computed Tomography, Society for Cardiovascular Magnetic Resonance (2011). ACCF/ASE/AHA/ASNC/HFSA/HRS/SCAI/SCCM/SCCT/SCMR 2011 Appropriate Use Criteria for Echocardiography. A Report of the American College of Cardiology Foundation Appropriate Use Criteria Task Force, American Society of Echocardiography, American Heart Association, American Society of Nuclear Cardiology, Heart Failure Society of America, Heart Rhythm Society, Society for Cardiovascular Angiography and Interventions, Society of Critical Care Medicine, Society of Cardiovascular Computed Tomography, Society for Cardiovascular Magnetic Resonance American College of Chest Physicians. J. Am. Soc. Echocardiogr..

[6] Steeds R.P., Garbi M., Cardim N., Kasprzak J.D., Sade E., Nihoyannopoulos P., Popescu B.A., Stefanidis A., Cosyns B., Monaghan M. (2017). EACVI appropriateness criteria for the use of transthoracic echocardiography in adults: A report of literature and current practice review. Eur. Heart J. Cardiovasc. Imaging.

[7] Barbieri A., Camilli M., Bisceglia I., Mantovani F., Ciampi Q., Zito C., Canale M.L., Khoury G., Antonini-Canterin F., Carerj S. (2024). Current use of echocardiography in cardio-oncology: Nationwide real-world data from an ANMCO/SIECVI joint survey. Eur. Heart J. Imaging Methods Pract..

[8] Barbieri A., Mantovani F., Ciampi Q., Barchitta A., Faganello G., Miceli S., Parato V.M., Tota A., Trocino G., Antonini-Canterin F. (2023). Current national availability of advanced echocardiography imaging: Real world data from an Italian Society of Echocardiography and Cardiovascular Imaging survey. Eur. Heart J. Imaging Methods Pract..

[9] Ciampi Q., Pepi M., Antonini-Canterin F., Barbieri A., Barchitta A., Faganello G., Miceli S., Parato V.M., Tota A., Trocino G. (2023). Organization and Activity of Italian Echocardiographic Laboratories: A Survey of the Italian Society of Echocardiography and Cardiovascular Imaging. J. Cardiovasc. Echogr..

[10] Ciampi Q., Antonini-Canterin F., Barbieri A., Barchitta A., Benedetto F., Cresti A., Miceli S., Monte I., Picano E., Pepi M. (2021). Remodeling of activities of Italian echocardiographic laboratories during the coronavirus disease 2019 lockdown: The SIECoVId study. J. Cardiovasc. Med..

[11] Ciampi Q., Antonini-Canterin F., Barbieri A., Barchitta A., Benedetto F., Cresti A., Miceli S., Monte I., Petrella L., Trocino G. (2021). Reshaping of Italian Echocardiographic Laboratories Activities during the Second Wave of COVID-19 Pandemic and Expectations for the Post-Pandemic Era. J. Clin. Med..

[12] Ciampi Q., Pepi M., Antonini-Canterin F., Barbieri A., Barchitta A., Faganello G., Miceli S., Parato V.M., Trocino G., Abbate M. (2023). Stress Echocardiography in Italian Echocardiographic Laboratories: A Survey of the Italian Society of Echocardiography and Cardiovascular Imaging. J. Cardiovasc. Echogr..

[13] Emilia-Romagna Region, Italy: Regional Health Profile. WHO Regions for Health Network 2022. https://cdn.who.int/media/docs/librariesprovider2/regions-for-health/2022-rhn-region-profiles-emilia-romagna.pdf.

[14] HIMSS (2026). HIMSS22 Europe Celebrates Healthcare Systems That Have Been Validated at EMRAM Stages 6 and 7. https://iowa.himss.org/news/himss22-europe-celebrates-healthcare-systems-validated-emram-stages-6-and-7.

[15] Odone A., Azzopardi-Muscat N. (2019). Health and the effect of universal health coverage in Italy. Lancet Public Health.

[16] Fascicolo Sanitario Elettronico–Ministero Della Salute 2026. https://www.fascicolosanitario.gov.it/portale/home.

[17] Popescu B.A., Stefanidis A., Nihoyannopoulos P., Fox K.F., Ray S., Cardim N., Rigo F., Badano L.P., Fraser A.G., Pinto F. (2014). Updated standards and processes for accreditation of echocardiographic laboratories from The European Association of Cardiovascular Imaging. Eur. Heart J. Cardiovasc. Imaging.

[18] Wiegers S.E., Ryan T., Arrighi J.A., Brown S.M., Canaday B., Damp J.B., Diaz-Gomez J.L., Figueredo V.M., Garcia M.J., Gillam L.D. (2019). 2019 ACC/AHA/ASE Advanced Training Statement on Echocardiography (Revision of the 2003 ACC/AHA Clinical Competence Statement on Echocardiography): A Report of the ACC Competency Management Committee. J. Am. Coll. Cardiol..

[19] Tang G.H.L., Zaid S., Hahn R.T., Aggarwal V., Alkhouli M., Aman E., Berti S., Chandrashekhar Y.S., Chadderdon S.M., D’Agostino A. (2025). Structural Heart Imaging Using 3-Dimensional Intracardiac Echocardiography: JACC: Cardiovascular Imaging Position Statement. JACC Cardiovasc. Imaging.

[20] Robinson S., Rana B., Oxborough D., Steeds R., Monaghan M., Stout M., Pearce K., Harkness A., Ring L., Paton M. (2020). A practical guideline for performing a comprehensive transthoracic echocardiogram in adults: The British Society of Echocardiography minimum dataset. Echo Res. Pract..

[21] Senior R., Becher H., Monaghan M., Agati L., Zamorano J., Vanoverschelde J.L., Nihoyannopoulos P., Edvardsen T., Lancellotti P., Delgado V. (2017). Clinical practice of contrast echocardiography: Recommendation by the European Association of Cardiovascular Imaging (EACVI) 2017. Eur. Heart J. Cardiovasc. Imaging.

[22] Picano E., Pierard L., Peteiro J., Djordjevic-Dikic A., Sade L.E., Cortigiani L., Van De Heyning C.M., Celutkiene J., Gaibazzi N., Ciampi Q. (2024). The clinical use of stress echocardiography in chronic coronary syndromes and beyond coronary artery disease: A clinical consensus statement from the European Association of Cardiovascular Imaging of the ESC. Eur. Heart J. Cardiovasc. Imaging.

[23] Lancellotti P., Pellikka P.A., Budts W., Chaudhry F.A., Donal E., Dulgheru R., Edvardsen T., Garbi M., Ha J.-W., Kane G.C. (2016). The clinical use of stress echocardiog;raphy in non-ischaemic heart disease: Recommendations from the European Association of Cardiovascular Imaging and the American Society of Echocardiography. Eur. Heart J. Cardiovasc. Imaging.

[24] ALQahtani D.A., Rotgans J.I., Mamede S., ALAlwan I., Magzoub M.E., Altayeb F.M., Mohamedani M.A., Schmidt H.G. (2016). Does Time Pressure Have a Negative Effect on Diagnostic Accuracy?. Acad. Med..

[25] Iskander J., Kelada P., Rashad L., Massoud D., Afdal P., Abdelmassih A.F. (2022). Advanced Echocardiography Techniques: The Future Stethoscope of Systemic Diseases. Curr. Probl. Cardiol..

[26] Monte I.P., De Chiara B., Demicheli G., Aragona P., Ancona R., Antonini-Canterin F., Citro R., Colonna P., Giorgi M., Mantero A. (2019). Update on the Organizational Aspects of Echocardiography in Italy (From Operator Training to the Report: 2007–2019): A Consensus Document by the “Societa Italiana di Ecocardiografia e CardioVascular Imaging” Accreditation Area and Board 2017–2019. J. Cardiovasc. Echogr..

[27] Mantero A., Gentile F., Alberti A., Bencini C., Bongarzoni A., Bragato R., Branzi G., Casazza F., Cialfi A., Dihel L. (2008). The “Appropriatezza ECO Milano” project. Assessment of appropriateness of indications, prescriptions and clinical utility of two-dimensional Doppler echocardiography among inpatients and outpatients of Milan, Italy. G. Ital. Cardiol..

[28] Lattanzi F., Magnani M., Cortigiani L., Mandorla S., Zuppiroli A., Lorenzoni R., Anmco-Toscana G.D.V.D. (2002). Evaluation of the appropriateness of prescribing echocardiography. Ital. Heart J. Suppl..

[29] Orsini E., Antoncecchi E., Carbone V., Dato A., Monducci I., Nistri S., Zito G.B., Study IN-opAARCAim (2013). Indications, Utility and Appropriateness of Echocardiography in Outpatient Cardiology. J. Cardiovasc. Echogr..

[30] Douglas P.S. (2012). Appropriate use criteria: Past, present, future. J. Am. Soc. Echocardiogr..

[31] Koshy T.P., Rohatgi A., Das S.R., Price A.L., deLuna A., Reimold N., Willett K., Reimold S.C., Matulevicius S.A. (2015). The association of abnormal findings on transthoracic echocardiography with 2011 Appropriate Use Criteria and clinical impact. Int. J. Cardiovasc. Imaging.

[32] Kerley R.N., O’Flynn S. (2019). A systematic review of Appropriate Use Criteria for transthoracic echocardiography: Are they relevant outside the United States?. Ir. J. Med. Sci..

[33] Ajmone Marsan N., Michalski B., Cameli M., Podlesnikar T., Manka R., Sitges M., Dweck M.R., Haugaa K.H. (2020). EACVI survey on standardization of cardiac chambers quantification by transthoracic echocardiography. Eur. Heart J. Cardiovasc. Imaging.

[34] Soliman-Aboumarie H., Joshi S.S., Cameli M., Michalski B., Manka R., Haugaa K., Demirkiran A., Podlesnikar T., Jurcut R., Muraru D. (2022). EACVI survey on the multi-modality imaging assessment of the right heart. Eur. Heart J. Cardiovasc. Imaging.

[35] Corbett L., O’Driscoll P., Paton M., Oxborough D., Surkova E. (2024). Role and application of three-dimensional transthoracic echocardiography in the assessment of left and right ventricular volumes and ejection fraction: A UK nationwide survey. Echo Res. Pract..

[36] Dey D., Slomka P.J., Leeson P., Comaniciu D., Shrestha S., Sengupta P.P., Marwick T.H. (2019). Artificial Intelligence in Cardiovascular Imaging: JACC State-of-the-Art Review. J. Am. Coll. Cardiol..

[37] Schuuring M.J., Isgum I., Cosyns B., Chamuleau S.A.J., Bouma B.J. (2021). Routine Echocardiography and Artificial Intelligence Solutions. Front. Cardiovasc. Med..

[38] Wong B.L.H., Maass L., Vodden A., van Kessel R., Sorbello S., Buttigieg S., Odone A., European Public Health Association (EUPHA) Digital Health Section (2022). The dawn of digital public health in Europe: Implications for public health policy and practice. Lancet Reg. Health Eur..

[39] Ward R.P., Mansour I.N., Lemieux N., Gera N., Mehta R., Lang R.M. (2008). Prospective evaluation of the clinical application of the American College of Cardiology Foundation/American Society of Echocardiography Appropriateness Criteria for transthoracic echocardiography. JACC Cardiovasc. Imaging.

[40] King S.J., Williamson C., Weickert T.P., Miller P.F., Hinderliter A.L., Stouffer G.A. (2024). Applicability of Appropriate Use Criteria for Echocardiography in an Underserved Population. J. Am. Soc. Echocardiogr..

[41] PNRR-Salute Piano Nazionale di Ripresa e Resilienza 2026. https://www.pnrr.salute.gov.it/it/pnrr-pagina/piano-nazionale-di-ripresa-e-resilienza-cose-la-missione-salute/.

