# Supplementary material for: Mapping Echocardiographic Practice in Emilia-Romagna: A Regional Healthcare Census
Source: J Clin Med. 2026 May 12;15(10):3719. doi: 10.3390/jcm15103719 (PMC13206855; doi:10.3390/jcm15103719)
Supplement: Supplementary file 1 [file jcm-15-03719-s001.zip › jcm-4203448-Supplementary Material S1 list .pdf]

Mapping Echocardiographic Practice in Emilia-Romagna: A Regional Healthcare Census

Supplementary Material 2-List of Facilities

- ACTF Bianalisi S. Ilario d’Enza, *Sant’Ilario d’Enza* (PR)*
- Aesculapio, *San Felice sul Panaro* (MO)
- Alliance Medical Carpi, *Carpi* (MO)
- AOU di Bologna - Policlinico di Sant’Orsola, *Bologna* (BO)
- AUSL Bologna - Outpatient Cardiology Services, *Bologna* (BO)
- AUSL Ferrara - Outpatient Cardiology Services, *Ferrara* (FE)
- AUSL Romagna - Outpatient Cardiology Services, multiple sites (FC)
- B.P.C. - Outpatient Clinic, *Maranello* (MO)
- C.A.F. - Outpatient Clinic, *Modena* (MO)
- C.F.T. - Outpatient Clinic, *Vignola* (MO)
- Cardiology Division, AOU di Ferrara - Arcispedale Sant’Anna di Cona, *Ferrara* (FE)
- Cardiology Division, AOU di Modena - Ospedale di Baggiovara, *Modena* (MO)
- Cardiology Division, AOU di Modena - Policlinico di Modena, *Modena* (MO)
- Cardiology Division, AOU di Parma, *Parma* (PR)
- Cardiology Division, Arcispedale Santa Maria Nuova, *Reggio Emilia* (RE)
- Cardiology Division, AUSL di Bologna - Ospedale Bellaria, *Bologna* (BO)
- Cardiology Division, AUSL di Bologna - UOS Cardiologia dell’Appennino, *Castiglione dei Pepoli* (BO)
- Cardiology Division, AUSL di Ferrara - Ospedale del Delta, *Lagosanto* (FE)
- Cardiology Division, AUSL di Ferrara - Ospedale Santissima Annunziata, *Cento* (FE)
- Cardiology Division, AUSL di Imola, *Imola* (BO)
- Cardiology Division, AUSL di Modena - Ospedale di Carpi, *Carpi* (MO)
- Cardiology Division, AUSL di Modena - Ospedale di Mirandola, *Mirandola* (MO)
- Cardiology Division, AUSL di Modena - Ospedale di Pavullo nel Frignano, *Pavullo nel Frignano* (MO)
- Cardiology Division, AUSL di Parma - Coronary Care Unit, *Parma* (PR)
- Cardiology Division, AUSL di Piacenza - Ospedale Guglielmo da Saliceto, *Piacenza* (PC)

- Cardiology Division, AUSL di Reggio Emilia - Ospedale di Guastalla, *Guastalla* (RE)
- Cardiology Division, AUSL Romagna - Ospedale Bufalini, *Cesena* (FC)
- Cardiology Division, AUSL Romagna - Ospedale di Faenza, *Faenza* (RA)
- Cardiology Division, AUSL Romagna - Ospedale di Lugo, *Lugo* (RA)
- Cardiology Division, AUSL Romagna - Ospedale di Riccione, *Riccione* (RN)
- Cardiology Division, AUSL Romagna - Ospedale Infermi di Rimini, *Rimini* (RN)
- Cardiology Division, AUSL Romagna - Ospedale Morgagni–Pierantoni, *Forlì* (FC)
- Cardiology Division, Hesperia Hospital, *Modena* (MO)
- Cardiology Division, Maria Cecilia Hospital, *Cotignola* (RA)
- Cardiology Division, Ospedale di Bentivoglio, *Bentivoglio* (BO)
- Cardiology Division, Ospedale di Sassuolo, *Sassuolo* (MO)
- Cardiology Division, Ospedale Giuseppe Dozzetti, *Bazzano* (BO)
- Cardiology Division, Ospedale Maggiore, *Bologna* (BO)
- Cardiology Service, AUSL di Bologna, multiple sites (BO)
- Cardiovascular Medicine Unit, Arcispedale Santa Maria Nuova, *Reggio Emilia* (RE)
- Casa della Comunità (Borgo Reno) - Outpatient Clinic, *Bologna* (BO)
- Casa della Comunità (Navile) - Outpatient Clinic, *Bologna* (BO)
- Casa della Comunità Cimarosa - Multiservice Facility, *Casalecchio di Reno* (BO)
- Casa della Comunità di Castelfranco Emilia - Outpatient Clinic, *Castelfranco Emilia* (MO)
- Casa della Comunità - Outpatient Clinic, *Casalecchio di Reno* (BO)
- Casa della Comunità - Outpatient Clinic, *San Lazzaro di Savena* (BO)
- Casa della Salute, *Castiglione dei Pepoli* (BO)
- Casa di Cura Città di Parma - Outpatient Clinic, *Parma* (PR)
- Casa di Cura Fogliani, *Modena* (MO)
- Casa di Cura Prof. Nobili - Outpatient Clinic, *Castiglione dei Pepoli* (BO)
- Casa di Cura Prof.E. Montanari, *Morciano di Romagna* (RN)
- Casa di Cura Quisisana, *Ferrara* (FE)
- Casa di Cura S. Antonino, *Piacenza* (PC)
- Casa di Cura S. Lorenzino, *Cesena* (FC)
- Casa di Cura Val Parma, *Langhirano* (PR)
- Casa di Cura Villa Verde, *Reggio Emilia* (RE)
- Centro Fisiolaserterapeutico Emiliano - Outpatient Clinic, *Ozzano dell'Emilia* (BO)
- Centro Fisioterapico Maria Luigia - Outpatient Clinic, *Parma* (PR)

- Centro Hercolani - Outpatient Clinic, *Bologna* (BO)
- Centro Medico Soteria - Outpatient Clinic, *Felino* (PR)
- Centro PASCIA, Cardiology Division, Policlinico di Modena, *Modena* (MO)
- Cerba HealthCare - Outpatient Clinic, *Imola* (BO)
- Cerba HealthCare - Outpatient Clinic, *Misano* (RN)
- Coliseum Center - Outpatient Clinic, *Modena* (MO)
- F Medical - Outpatient Clinic, *Ferrara* (FE)
- Fisiomedical - Outpatient Clinic, multiple sites (MO)
- Fondazione Don Gnocchi - Cardiovascular Prevention and Rehabilitation Unit, *Parma* (PR)
- Hesperia Diagnostic Center - Outpatient Clinic, *Carpi* (MO)
- Ionoforetica - Outpatient Clinic, *Bologna* (BO)
- Medical Thermae - Outpatient Clinic, *Sant'Andrea Bagni* (PR)
- Medipass - Outpatient Clinic, *Crevalcore* (BO)
- Ospedale Cervesi - Cardiology Division, *Cattolica* (RN)
- Ospedale di Borgotaro, *Borgo Val di Taro* (PR)
- Ospedale di Castel San Giovanni, *Castel San Giovanni* (PC)
- Ospedale E. Franchini di Montecchio Emilia, *Montecchio Emilia* (RE)
- Ospedale Franchini di Santarcangelo di Romagna, *Santarcangelo di Romagna* (RN)
- Ospedale Privato Salus, *Ferrara* (FE)
- Ospedali Privati Forlì, *Forlì* (FC)
- Outpatient Cardiology Clinic, AUSL di Modena - Vignola, *Vignola* (MO)
- Outpatient Clinic of Sports and Cardiovascular Medicine, *Cesena* (FC)
- Pediatric Cardiology Division, AOU di Bologna - Policlinico di Sant'Orsola, *Bologna* (BO)
- Pediatric Cardiology Division, AOU di Parma, *Parma* (PR)
- Piccole Figlie Hospital, *Parma* (PR)
- Poliambulatorio Cavour - Outpatient Clinic, *Sasso Marconi* (BO)
- Poliambulatorio Centro Integrato Neuroscienze - Outpatient Clinic, *Rimini* (RN)
- Poliambulatorio Chersich - Outpatient Clinic, *Bologna* (BO)
- Poliambulatorio Comedent - Outpatient Clinic, *Modena* (MO)
- Poliambulatorio Descovich - Outpatient Clinic, *Bologna* (BO)
- Poliambulatorio La Salute - Outpatient Clinic, *Anzola dell'Emilia* (BO)
- Poliambulatorio Medica - Outpatient Clinic, *San Giovanni in Persiceto* (BO)
- Poliambulatorio Mengoli - Outpatient Clinic, *Bologna* (BO)

- Poliambulatorio Reno - Outpatient Clinic, *Bologna* (BO)
- Poliambulatorio San Camillo - Outpatient Clinic, *Bologna* (BO)
- Poliambulatorio Test - Outpatient Clinic, *Modena* (MO)
- Ravenna Medical Center - Outpatient Clinic, *Ravenna* (RA)
- Ravenna33 - Outpatient Clinic, *Ravenna* (RA)
- Riminiterme - Outpatient Clinic, *Rimini* (RN)
- Salus Hospital, *Reggio Emilia* (RE)
- San Giuseppe - Outpatient Clinic, *Zola Predosa* (BO)
- San Pier Damiano Hospital, *Faenza* (RA)
- Sol et Salus Hospital, *Torre Pedrera* (RN)
- Terme della Salvarola - Outpatient Clinic, *Sassuolo* (MO)
- Terme di Punta Marina - Outpatient Clinic, *Punta Marina* (RA)
- Territorial Cardiology Service, AUSL di Parma, *Parma* (PR)
- Villa Laura Hospital, *Bologna* (BO)
- Villa Pineta, *Pavullo nel Frignano* (MO)
- Villa Torri Hospital, *Bologna* (BO)

**Physically located in Sant'Ilario d'Enza (RE), administratively referring to AUSL Parma.*

Notes

Facilities are listed in alphabetical order. Each participating center was defined as an organizational unit independently delivering echocardiographic services. Multiple centers operating within the same hospital or facility were considered as distinct units when separate survey entries were completed. For reporting purposes, outpatient services managed by the same Local Health Authority within a given province were grouped and reported as a single center. This approach was adopted to improve clarity and readability of the center registry and did not affect quantitative analyses.

Province abbreviations are reported according to the Italian administrative system *(BO = Bologna; FC = Forlì-Cesena; FE = Ferrara; MO = Modena; PR = Parma; PC = Piacenza; RA = Ravenna; RE = Reggio Emilia; RN = Rimini).*
