# Supplementary material for: Mapping Echocardiographic Practice in Emilia-Romagna: A Regional Healthcare Census
Source: J Clin Med. 2026 May 12;15(10):3719. doi: 10.3390/jcm15103719 (PMC13206855; doi:10.3390/jcm15103719)
Supplement: Supplementary file 1 [file jcm-15-03719-s001.zip › jcm-4203448-Supplementary Material S2 update.pdf]

Comprehensive Regional Census of Echocardiography Laboratories: The Emilia-Romagna SIECVI Survey

Supplementary Material 2

Full Questionnaire

1) The collection of information through the questionnaire is aimed exclusively at statistical processing, also carried out with electronic tools by the SIECVI to receive information on the current situation of ultrasound laboratories and to imagine possible implementations to facilitate the work of sonographers. The data collected may also be used by some research group members or for other scientific purposes. THE DATA REFER TO THE ACTIVITY CARRIED OUT IN THE PERIOD 01/01/2023-31/12/2023. The data will be processed in compliance with the Code regarding personal data protection (legislative decree 196/03). I consent to the use of data Yes/No

2) Age of the respondent: <35 / 36–50 / 51–65 / >65

3) Gender of the respondent: Male / Female / Prefer not to specify

4) Type of facility: Territorial Cardiology / Spoke / Hub Center / Hub with Cardiac Surgery / Hub with Cardiac Surgery and Transplant Unit / Accredited Private Center

5) Name of the facility

6) Email address for follow-up contact (if needed)

7) Province where the activity is primarily carried out

8) Is this a university training center? Yes – Main / Yes – Branch / No

9) Is the laboratory accredited by SIECVI and/or EACVI? Yes / No

10) Is there a designated responsible (unit chief/high-specialty lead)? Yes / No

11) If not, is there an official point of reference? Yes / No

12) Number of boxes/offices composing the echocardiographic lab

13) Number of cardiologists on the team

14) Number of sonographers on the team

15) Is telecardiology performed via data archiving systems (RIS/PACS) if working with sonographers? Yes / No

16) Number of top-level ultrasound machines (with 3D probes and advanced software)

17) Number of mid-level ultrasound machines

18) Number of portable ultrasound machines

19) Number of handheld ultrasound devices

20) How much time does the team dedicate to imaging activities? Predominantly (>75%) / Majority (50–75%) / Minor (25–50%) / Occasionally (<25%)

21) Does the lab have an image archiving system? No / Yes on PACS / Yes on external archive (CD, DVD, USB)

22) Is there a dedicated reporting system? No / Yes, with free-text reporting / Yes, with structured reporting

23) Is digital signature and electronic health record archiving available? Yes / No / Not yet, but being implemented

24) Does the lab perform outpatient services booked through the CUP system? No / Yes (<20%) / Yes (20–50%) / Yes (>50%)

25) Average monthly number of transthoracic echocardiograms (based on 2023 annual volume divided by 12).

26) Are supra-aortic vessels Doppler ultrasounds performed? Yes / No

27) Monthly number of supra-aortic vessels Doppler exams

28) Are bubble contrast echocardiograms performed? Yes / No

29) Monthly number of bubble contrast echocardiograms

30) Are contrast-enhanced echocardiograms performed? Yes / No

31) Monthly number of contrast-enhanced echocardiograms

32) Is a treadmill or bicycle ergometer available for stress echo? Yes / No

33) Is exercise stress echocardiography performed? Yes / No

34) Monthly number of exercise stress echocardiography

35) Is dipyridamole stress echocardiography performed? Yes / No

36) Monthly number of dipyridamole stress echocardiography

37) Is coronary flow reserve assessment performed? Yes / No

38) Is dobutamine stress echocardiography performed? Yes / No

39) Monthly number of dobutamine stress echocardiography

40) Is transcranial Doppler ultrasound performed? Yes / No

41) Monthly number of transcranial Doppler exams

42) Is pediatric TTE performed? Yes / No

43) If yes, which age range: <4 weeks (neonatal) / 4 weeks–3 years / 3–6 years / >6 years / All age groups

44) Monthly number of pediatric echocardiograms

45) Is TEE performed? Yes / No

46) Monthly number of TEE

47) Is 3D TEE analysis software available? Yes on ultrasound machine / Yes on dedicated workstation / No

48) Is echocardiographic support provided for structural procedures? Yes / No

49) If yes, for which procedures? LAA closure / TAVI / Percutaneous mitral valve procedures / Percutaneous tricuspid valve procedures / Other

50) Is 3D transthoracic LV analysis software available? Yes on ultrasound machine / Yes on dedicated workstation / No

51) If yes, the analysis is automatic/semi-automatic

52) Is 3D transthoracic RV analysis software available? Yes on ultrasound machine / Yes on dedicated workstation / No

53) If yes, the analysis is automatic/semi-automatic

54) Is LV strain analysis software available? Yes on ultrasound machine / Yes on dedicated workstation / No

55) If yes, the analysis is automatic/semi-automatic

56) Is RV strain analysis software available? Yes on ultrasound machine / Yes on dedicated workstation / No

57) If yes, the analysis is automatic/semi-automatic

58) Is LA strain analysis software available? Yes on ultrasound machine / Yes on dedicated workstation / No

59) If yes, the analysis is automatic/semi-automatic

60) Is RA strain analysis software available? Yes on ultrasound machine / Yes on dedicated workstation / No

61) If yes, the analysis is automatic/semi-automatic

62) How long is it estimated to perform TTE? 10′/15′/20′/30′/40′/45′/60′

63) How long is it estimated to perform TEE? 10′/15′/20′/30′/40′/45′/60′

64) How long is it estimated to perform exercise stress echocardiography? 10′/15′/20′/30′/40′/45′/60′

65) How long is it estimated to perform pharmacological stress echocardiography? 10′/15′/20′/30′/40′/45′/60′

66) How long is it estimated to perform TTE + echo contrast? 10′/15′/20′/30′/40′/45′/60′

67) To what extent do you consider the indications that reach your echocardiography laboratory inappropriate? 10%/25%/50%/75%/90%

68) What are, in your opinion, the main critical issues of your laboratory? Inadequate technological equipment, poor appropriateness in the tests requested/Shortage of dedicated personnel/Inadequate timing/Poor access to echocardiographic training courses/other - specify below

69) other specification

70) Are you a member of any scientific societies? Yes/No

71) If so, which ones?

72) What would you like to see implemented by scientific societies in your region? Training courses in basic and transthoracic echocardiography/Training courses on advanced echocardiography methods (3D, strain)/Live or online meetings with analysis of clinical case/Meetings with discussion on the paths that converge on cardiovascular imaging/Comparison meetings on the organization of the echo lab/Other – Specify

73) Specification

74) How many regional cardiac imaging scientific events would you be available to attend in person each year? 1/2/3/4/None

75) How many national/international imaging conferences have you attended in the last 10 years? 1/2/3/4/5/5+

Table. Sensitivity analyses using alternative definitions of structural–digital readiness.

Predictor	OR (score = 5)	95% CI	p	OR (score ≥3)	95% CI	p
Teaching status (University main vs no)	10.9	1.2–134.1	**0.033**	9.7	1.07–248	**0.042**
Teaching status (University branch vs no)	17.9	3.2-156.5	**0.0007**	2.01	0.56-7.82	0.285
Designated echocardiography lead	2.1	0.5–5.05	0.29	3.43	1.18–10.9	**0.023**
Public vs. private	1.4	0.2–11.8	0.730	1.9	0.7–4.9	0.07
Volume	0.94	0.52–1.75	0.34	1.93	1.19–3.29	**0.007**

Sensitivity analyses exploring alternative operational definitions of the structural–digital readiness index. Multivariable logistic regression models were repeated using a stringent definition (score = 5) and a more permissive definition (score ≥ 3). Procedural volume was entered as the natural logarithm of the average monthly number of transthoracic echocardiograms. Results are reported as odds ratios (ORs) with 95% confidence intervals (CIs).

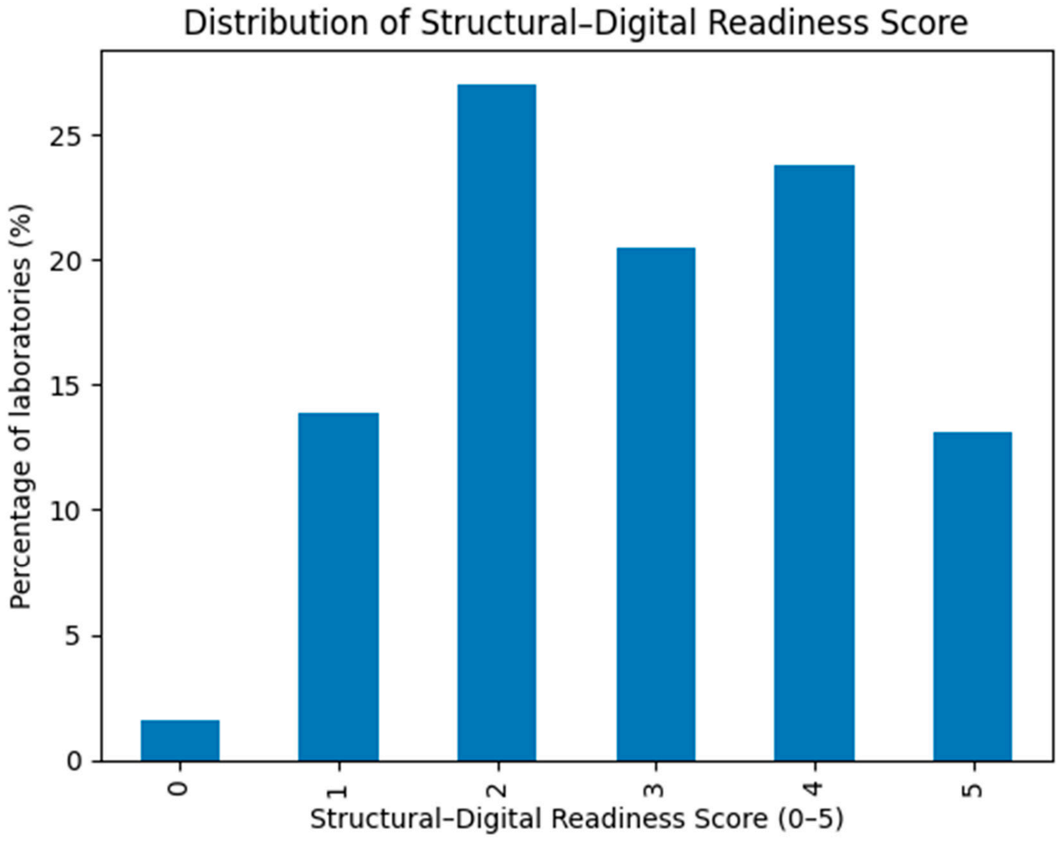

Figure. Distribution of the structural-digital readiness index (0–5) across echocardiography laboratories.
